# 

**Published:** 1894-04

**Authors:** 


### CONTENTS—APRIL, 1894.—VOL. IX., NO. 10.

ORIGINAL COMMUNICATIONS—
Ethical Relationship of the Medical Profession and Life Insurance Companies. S. H. Stout, M. D., Dallas . . . . . . . . . . . . . . 493
Some Observations of Thiersch's Method of Skin Grafting, Illustrated by Two Cases Shown. William Gammon, M. D. . . . . . . . . 498
Was It Hydrophobia? Drs. Brown and Baily, Richland. . . . . . . 502
The Mastoid Operation. B. F. Church, M. D., Terrell . . . . . . . . 503
Report of a Case of Outward Dislocation of the Knee. T. J. Bennett, M. D., Austin . . . . . . . . . . . . . . . . . . . . . . . . 505
Umbilical Hemorrhage. Thos. B. Grayson, M. D., Winkler . . . . 507
ABSTRACTS OF CURRENT MEDICAL LITERATURE—
Department of Therapeutics. Edited by Prof. D. Cerna, M. D., Galveston . . . . . . . . . . . . . . . . . . . . . . . . . . . . . 508
Department of Gynecology. Edited by Prof. Wm. Keiller, F. R. C. S., Ed., Galveston . . . . . . . . . . . . . . . . . . . . . . . . . . 511
Department of Practice of Medicine. Edited by Prof. A. J. Smith, M. D., Galveston. . . . . . . . . . . . . . . . . . . . . . . . . . 515
SOCIETY NOTES—
Texas State Medical Association—Program . . . . . . . . . . . . 519
Texas State Medical Association—Circular . . . . . . . . . . . . 525
The Medical Association . . . . . . . . . . . . . . . . . . . . . . . 525
EDITORIAL—
The Journal and the Association . . . . . . . . . . . . . . . . . 527
The Discovery of Ether Anæsthesia . . . . . . . . . . . . . . . . 530
Not Dreamt of in Horatio's Philosophy . . . . . . . . . . . . . . 533
Magnetic Healers and Sight Diagnosticians . . . . . . . . . . . 635
The Texas Medical Law . . . . . . . . . . . . . . . . . . . . . . . 537
United Confederate Veterans . . . . . . . . . . . . . . . . . . . . 538
A Whited Sepulchre . . . . . . . . . . . . . . . . . . . . . . . . . . 539
That Decision . . . . . . . . . . . . . . . . . . . . . . . . . . . . . 540
MEDICAL NEWS AND MISCELLANY—
Numerous News Items . . . . . . . . . . . . . . . . . . . . . . . . 540
BOOK NOTICES— . . . . . . . . . . . . . . . . . . . . . . . . . . . 544
PUBLISHERS' NOTES . . . . . . . . . . . . . . . . . . . . . . . . . 549

